# A *NOTCH1* Mutation Found in a Newly Established Ovarian Cancer Cell Line (FDOVL) Promotes Lymph Node Metastasis in Ovarian Cancer

**DOI:** 10.3390/ijms24065091

**Published:** 2023-03-07

**Authors:** Wei Jiang, Xueyan Ouyang, Chunjuan Jiang, Lina Yin, Qianlan Yao, Xuan Pei, Zhaodong Ji, Ming Li, Shaoli Song, Wentao Yang, Shenglin Huang, Huijuan Yang, Boer Shan

**Affiliations:** 1 Department of Gynecological Oncology, Fudan University Shanghai Cancer Center, Fudan University, Shanghai 200032, China; 2 Department of Oncology, Shanghai Medical College, Fudan University, Shanghai 200032, China; 3 Department of Nuclear Medicine, Fudan University Shanghai Cancer Center, Fudan University, Shanghai 200032, China; 4 Department of Pathology, Fudan University Shanghai Cancer Center, Fudan University, Shanghai 200032, China; 5 Department of Clinical Laboratory, Huashan Hospital, Fudan University, Shanghai 200040, China; 6 Cancer Institute, Fudan University Shanghai Cancer Center, Shanghai 200032, China

**Keywords:** *NOTCH1*-p.C702fs mutation, newly established ovarian cancer cell line (FDOVL), peritoneal and lymph node metastasis, ovarian cancer

## Abstract

Peritoneal implantation and lymph node metastasis have different driving mechanisms in ovarian cancer. Elucidating the underlying mechanism of lymph node metastasis is important for treatment outcomes. A new cell line, FDOVL, was established from a metastatic lymph node of a patient with primary platinum-resistant ovarian cancer and was then characterized. The effect of *NOTCH1*-p.C702fs mutation and NOTCH1 inhibitor on migration was evaluated in vitro and in vivo. Ten paired primary sites and metastatic lymph nodes were analyzed by RNA sequencing. The FDOVL cell line with serious karyotype abnormalities could be stably passaged and could be used to generated xenografts. *NOTCH1*-p.C702fs mutation was found exclusively in the FDOVL cell line and the metastatic lymph node. The mutation promoted migration and invasion in cell and animal models, and these effects were markedly repressed by the NOTCH inhibitor LY3039478. RNA sequencing confirmed CSF3 as the downstream effector of *NOTCH1* mutation. Furthermore, the mutation was significantly more common in metastatic lymph nodes than in other peritoneal metastases in 10 paired samples (60% vs. 20%). The study revealed that NOTCH1 mutation is probably a driver of lymph node metastasis in ovarian cancer, which offers new ideas for the treatment of ovarian cancer lymph node metastasis with NOTCH inhibitors.

## 1. Introduction

Ovarian cancer is the second most common cause of death related to gynecologic malignancy in women around the world [1]. It is estimated that a total of 225,500 cases of ovarian cancer will be diagnosed, and 140,200 patients will die of recurrent ovarian cancer and extensive metastasis each year [2]. Approximately 90% of ovarian cancers are epithelial ovarian cancer (EOC), and high-grade serous ovarian carcinoma (HGSOC) is the most common type of EOC. Because of its insidious onset and rapid progression, 75% of patients are diagnosed in an advanced stage (FIGO stage III/IV) accompanied by extensive intra-abdominal metastasis, and their 5-year overall survival rate is approximately 40% [3]. Standard first-line treatment relies on debulking surgery and platinum-based chemotherapy, followed by maintenance treatment with poly ADP-ribose polymerase inhibitor (PARPi) or anti-angiogenic agents according to BRCA1/2 status. Even though new strategies, such as the addition of HIPEC to interval cytoreductive surgery, prolonged recurrence-free survival and overall survival, most patients relapse within 18 months after their treatment ends [4]. Platinum sensitivity is an important determinant of prognosis. Platinum-resistant ovarian cancer, defined as ovarian cancer that relapses within 6 months after six to eight cycles of first-line platinum-based chemotherapy, is the most difficult type to treat. The establishment of a primary platinum-resistant ovarian cancer cell line would provide a good tool to fully understand the molecular biological behavior, which is of importance to improve the prognosis of ovarian cancer.

Lymph nodes are critical sites of distant metastasis in ovarian cancer. In cases of serous tumors, most lymph node metastases are located in the para-aortic region, even up the mediastinum to the clavicle, while for non-serous tumor, the rates of pelvic node and para-aortic node involvement are equal [5]. For patients with advanced-stage ovarian cancer, the rate of microscopic lymph node metastasis is reported to be more than 50% [6]. Recurrence at retroperitoneal lymph nodes is also common for ovarian cancer. In a retrospective study on recurrent ovarian cancer, the incidence of lymph node metastasis alone or combined with metastasis to other intraperitoneal sites was approximately 51.1% [7]. If metastatic lymph nodes reach the renal vasculature or more distant sites, the difficulty and risk of surgery will increase. If patients who have undergone more than two cytoreductive surgeries still relapse with lymph node metastasis, clinicians usually have no other choice but chemotherapy. However, chemotherapeutic drugs are inefficient for lymph node metastasis because of poor penetrability. Therefore, exploring the mechanism of lymph node metastasis in ovarian cancer and finding an applicable target will greatly improve the survival of ovarian cancer patients with lymph node metastasis.

In the present study, a new cell line, FDOVL, was established from a metastatic lymph node of a primary platinum-resistant ovarian cancer patient and was then characterized. *NOTCH1*-p.C702fs mutation was found exclusively in the FDOVL cell line and the metastatic lymph node. The mutation promoted migration and invasion at the cellular and animal levels, and these effects could be remarkably repressed by the NOTCH inhibitor LY3039478. Interestingly, the mutation could restore lymph node metastasis in animal models. RNA sequencing confirmed CSF3 as the downstream effector of *NOTCH1* mutation. Furthermore, the mutation was significantly more common in metastatic lymph nodes than in other peritoneal metastases in 10 paired samples. The establishment of FDOVL cells provides a good tool for exploring platinum resistance mechanisms in ovarian cancer. More importantly, this study revealed that *NOTCH1* mutation is probably a driver event of lymph node metastasis in ovarian cancer, which offers new thoughts for the treatment of ovarian cancer lymph node metastasis with NOTCH inhibitors.

## 2. Results

### 2.1. Morphology, Growth, Transplantation, and Genetic Characteristics of FDOVL Cells

**Figure 1:**
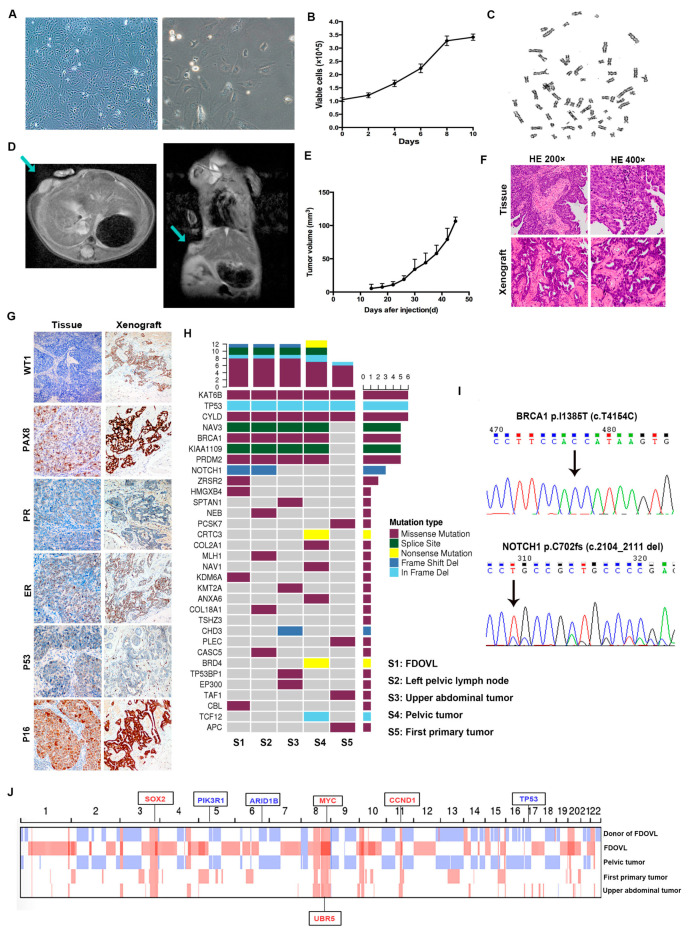
Morphology, growth, and transplantation and genetic characteristics of FDOVL cells. FDOVL cells stably passaged until 32 generation, then were subjected to the detection of general characteristics. (**A**) Phage contrast of microscopy (magnification 100× and 400×). (**B**) The grow curve of FDOVL cells. (**C**) Karyotype of FDOVL cells. (**D**–**F**) A total of 1 × 10^7^ FDOVL cells (32 generations) were used to establish in vivo xenograft tumor models in three nude mice (4 weeks, female). Magnetic resonance imaging (MRI) of transplanted tumors was performed 10 days after the tumor was visible to the naked eye. The representative images in the horizontal and coronal plane were shown. The tumor in the dorsal flank was pointed out by arrows (**D**). (**E**) The proliferation curve of transplanted tumor of FDOVL cells. (**F**) The transplanted tumors were surgically removed until the tumor volume reached 100 mm^3^, and then subjected to HE staining. (**G**) The molecular representation of ovarian cancer by IHC with the FDOVL cells and its donor tissue. The genomic information of FDOVL cells and the corresponding lymph node tissue, as well as the primary focality of the first and second debulking surgeries, including the upper abdominal and pelvic tumor, were compared by WES. (**H**) Heat map of driver genes variant was shown. Notably, *BRCA1* p.I1358T (c.T4254C) mutation was found in FDOVL cells and three tissue from the second, but the primary debulking surgery. *NOTCH1* p.C702fs (c.2104_2111 del) mutation was exclusively detected in FDOVL cells and its donor lymph node. (**I**) Sanger sequencing was used to confirm the mutation of *BRCA1* and *NOTCH1*. (**J**) Copy number variant (CNV) analysis of the five above samples was shown. The genes in red mean copy number gain, the genes in blue mean copy number loss.

The FDOVL cell line was passaged for more than 80 generations and was successfully recovered after freezing. It grew in the form of an adherent monolayer without contact inhibition and varied in shape (circular, elliptic, fusiform flat, or irregular) (Figure 1A). The FDOVL cell line was passaged routinely every four days. In contrast to the other common ovarian cancer cell lines, FDOVL cells do not proliferate rapidly, and the doubling time was 87 h. The growth curve is shown in Figure 1B. A total of 20 mitotic images and 10 karyotypes were obtained. FDOVL cells exhibited serious karyotype abnormalities, including ectopic recombination, mosaicism, and chromosome number abnormalities. The karyotypes of all mitotic phases were inconsistent, with 58–62 (90%) chromosomes, and the number of individual cells was greater than 80 (10%), which was consistent with that of malignant tumors (Figure 1C).

FDOVL cells were injected subcutaneously into three mice. All mice developed visible tumors 14 days after injection. Figure 1D presents the MRI image of transplanted tumors 24 days after transplantation. In addition, the growth curve of xenograft tumors is shown in Figure 1E. The xenografts and the primary tumor showed similar morphology according to HE staining (Figure 1F). The intensive positive staining of PAX8 and WT1 in xenografts indicated the origin of serous ovarian carcinoma (Figure 1G). In addition, the weak positive staining of P53 was consistent with the *TP53* mutation detected by WES.

WES was performed for FDOVL cells, the left pelvic lymph nodes, the upper abdominal tumor, the pelvic tumor from the second debulking surgery and the primary tumor from the first surgery. The above samples share some somatic cancer related single nucleotide variants (SNVs) of driver genes, including *KAT6B* (NM_001256468: exon 5: c.C845T: p.A282V), *TP53* (NM_001126115: exon 3: c.277_279 del: p.93_93del) and *CYLD* (NM_001042355: exon 9: c.A1577C: p.K526T) (Figure 1H). Interestingly, we detected some genetic variations exclusively in the samples from the second surgery but not those from the first surgery, such as *KIAA1109* (NM_015312: exon 59: c.10313-1G>T), *PRDM2* (NM_001007257: exon 3: c. C3716T: p. A1239V) and *BRCA1* (NM_007297: exon 11: c.T4154C:p.I1385T), indicating that the variations evolved from the treatment of ovarian cancer. In addition, both FDOVL cells and donor left pelvic lymph nodes presented frameshift deletion of *NOTCH1* (NM_017617: exon 13: c.2104_2111del: p.C702fs), but the other samples, including the upper abdominal and pelvic lesions from the secondary surgery and the primary tumors from the first surgery, did not. *BRCA1* and *NOTCH1* mutations were confirmed by Sanger sequencing (Figure 1I).

From Figure 1J, some common copy number variations (CNVs) were observed in samples from the first and secondary surgery, including amplified *CCND1* (11q13.3), *MYC* (8q24.21), *SOX2* (3q26.33), *UBR5* (8q22.3), deletion in *TP53* (17p13.1), *PIK3R1* (5q13.1), and *ARID1B* (6q25.3). In particular, the tissue from pelvic and left pelvic lymph nodes showed better similarity than that of upper abdominal tumors. Similarly, the four samples from secondary surgery shared some CNVs, while those variations were not detected in the primary tumor from the first surgery (Supplementary Table S4). For example, the *MAPK1* copy number increased in the tumor from the secondary surgery while no change was observed in the first surgery, and *ARID1A* deletion was detected in the samples from the secondary surgery rather than that from the first surgery. Interestingly, among the differentially expressed genes, we found that genes functioning in mismatch repair, such as *POLE*, *MSH2* and *MSH6*, and DNA damage repair, such as *BRCA2* and *ARID1B*, were defective in the samples from the secondary surgery but not in those from the first surgery.

### 2.2. NOTCH1-p.C720fs Mutation Promotes Epithelial-Mesenchymal Transition (EMT) and Its Effects Could Be Attenuated by a NOTCH1 Inhibitor in Ovarian Cancer Cells

**Figure 2:**
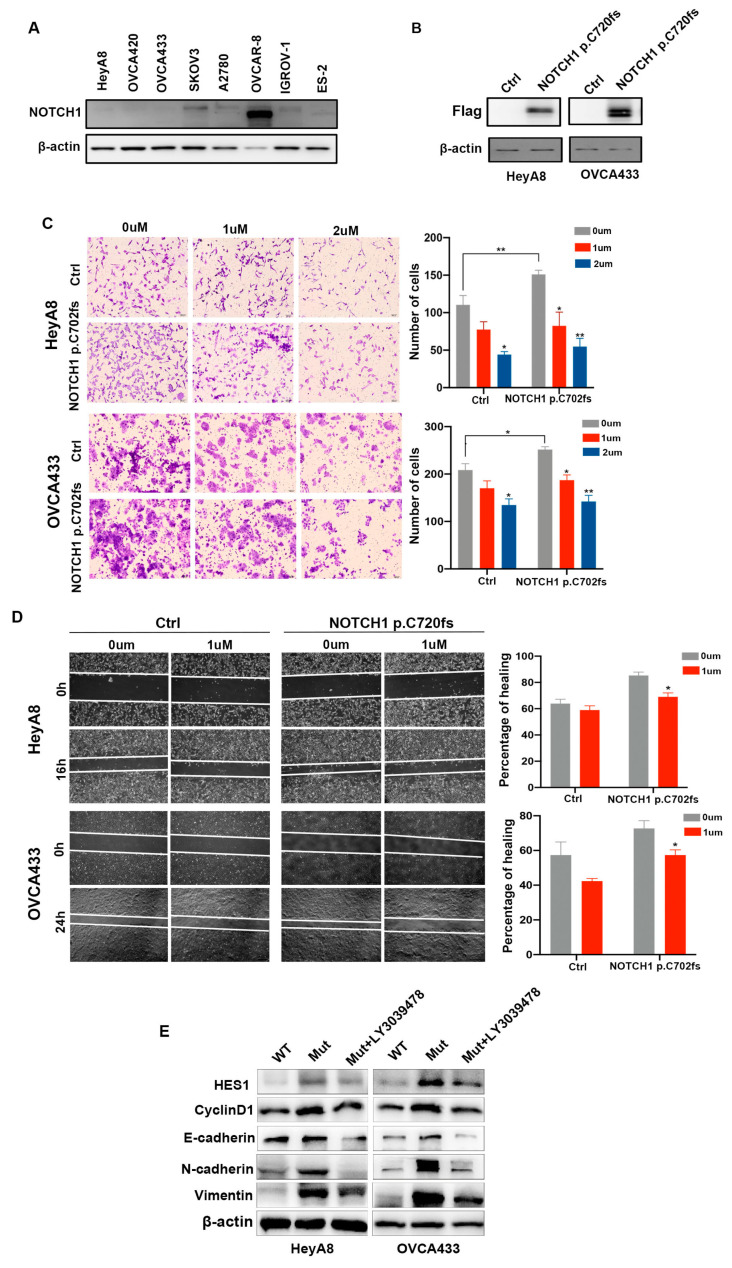
*NOTCH1*-p.C720fs mutation promotes epithelial-mesenchymal transition (EMT) and could be controlled by a NOTCH1 inhibitor in ovarian cancer cells. HeyA8 and OVCA433 cells were selected for the function of *NOTCH1*- p.C720fs mutation because of low expression of NOTCH1. An empty vector or that with the *NOTCH1* mutation were infected to HeyA8 and OVCA433 cells. HeyA8 and OVCA433 cells with wild-type and mutated Notch1 were pretreated with vehicle, 1 µM and 2 µM LY3039478 for 24 h, then subjected to transwell and wound healing assay to evaluate *NOTCH1*-p.C720fs mutation on migration and the efficacy of NOTCH inhibitor LY3039478. (**A**) The expression level of NOTCH1 NTM in nine ovarian cancer cell lines. (**B**) The expression of flag tag confirmed that HeyA8 and OVCA433 cells stably harbored *NOTCH1*-p.C720fs mutation by Western blot. (**C**–**E**) *NOTCH1*-p.C720fs mutation significantly enhances migratory ability, LY3039478 could inhibit migration in cells with wild-type and mutated NOTCH1 on the dependence of dose, and the inhibitory efficacy was more remarkable in cells with mutated NOTCH1. Migratory ability was analyzed by the transwell (**C**) and wound healing (**D**) assay, and the quantitative analysis of the migrated cells was showed on the left. (**E**) The expression of EMT and proliferation pathway related molecules showed corresponding change when the cells were pretreated with 2 µM of LY3039478. * *p* < 0.05, ** *p* < 0.01.

Next, we further explored whether the *NOTCH1* mutation (NM_017617: exon 13: c.2104_2111del: p.C702fs) found in metastatic lymph nodes could be related to metastasis in ovarian cancer. HeyA8 and OVCA433 cells were chosen because of the low expression of NOTCH1-NTM (Figure 2A). Lentivirus-carried Flag-NOTCH1 ^p.C720fs^ was transfected and verified (Figure 2B). Transwell and wound healing analyses were performed and compared between the control group and the *NOTCH1*-p.C720fs mutation. Moreover, the effect of NOTCH inhibition (LY3039478) was observed. Migration and invasion were significantly increased in HeyA8 and OVCA433 cells with NOTCH1 ^p.C720fs^ versus those without the mutation, and these effects were attenuated by LY3039478 in both the control and mutated groups; however, the inhibitory effect was more obvious in cells with NOTCH1 ^p.C720fs^ (Figure 2C,D). In addition, key metastasis-related molecules, such as E-cadherin and N-cadherin, exhibited corresponding changes (Figure 2E).

### 2.3. CSF3 Is Determined as the Downstream Factor by Which NOTCH1-p.C720fs Mutation Affects Epithelial-Mesenchymal Transition

**Figure 3:**
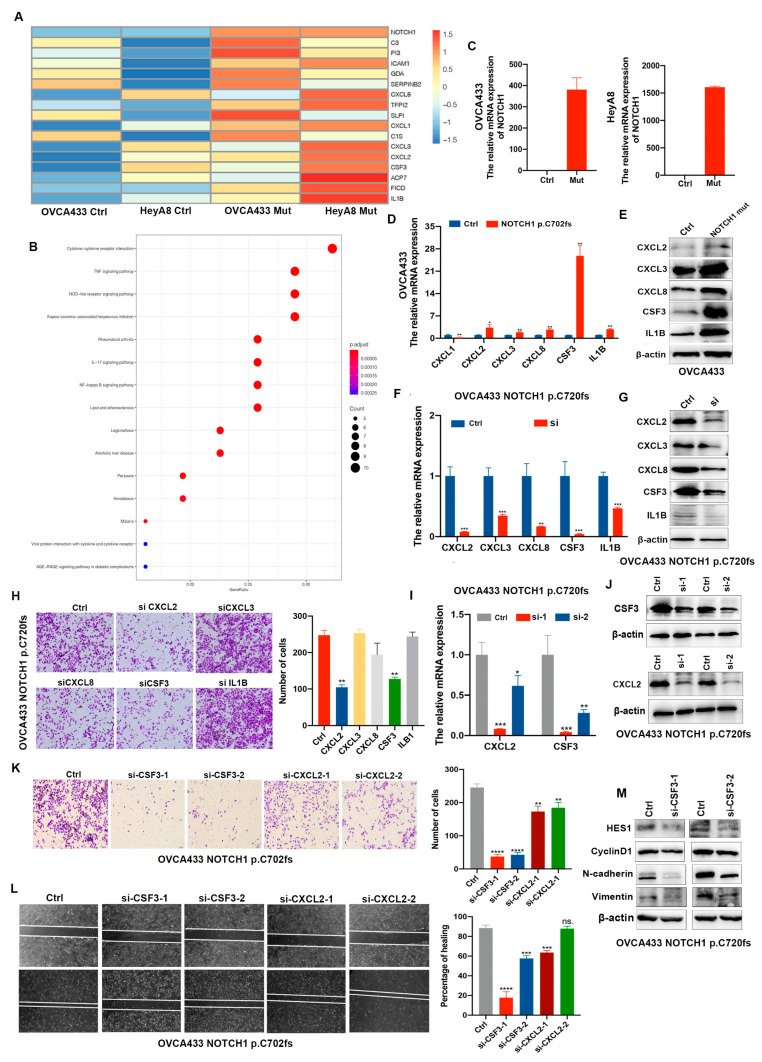
CSF3 is determined as the downstream effector of *NOTCH1*-p.C720fs mutation on epithelial-mesenchymal transition. (**A**,**B**) *CXCL1*, *CXCL2*, *CXCL3*, *CXCL8*, *CSF3* and *IL1B* were determined as the candidate genes regulated by the *NOTCH1* mutation. (**A**) The heat map of RNA sequence showed the differentially expressed genes between HeyA8, OVCA433 cells and those with *NOTCH1*-p.C720fs mutation. (**B**) The differentially expressed genes were analyzed by KEGG pathway. It showed that cytokine–cytokine receptor interaction ranked the first. (**C**) The mRNA expression of NOTCH1 in cells with mutated and wild-type NOTCH1 was confirmed by RT-PCR. The mRNA (**D**) and protein expression (**E**) of six candidates was detected in HeyA8, OVCA433 cells with mutated and wild-type NOTCH1, which narrows down the candidate genes to *CXCL1*, *CXCL2*, *CXCL3*, *CXCL8*, *CSF3* and *IL1B*. (**E**–**G**) Inhibition the expression of CSF3 or CXCL2 decreased the migratory ability in OVCA433 cells. OVCA433 cells were transfected with siRNA to reduce expression of the five candidates and verified by RT-PCR (**E**) and Western blot (**F**). (**G**) The migratory capability was detected by transwell assay. It showed that the migratory ability was inhibited when knocking down the expression of CSF3 or CXCL2. (**I**,**M**) Knocking down the expression of CSF3 reversed enhanced migration capability mediated by the *NOTCH1*-p.C720fs mutation. OVCA433 cells stably harboring mutated NOTCH1 were transfected with siRNA of CXCL2 and CSF3 (si-1 or si-2). QPCR (**F**) and Western blot (**G**) were used to verify the mRNA and protein expression level of CXCL2 and CSF3. (**H**,**I**) Depletion of CSF3 reverses the increased migratory ability caused by *NOTCH1*-p.C720fs mutation. OVCA433 cells transfected as described in F were subjected to transwell chamber (**H**) and wound healing assay (**I**) to assess the migration after 24 h incubation. The quantitative analysis was shown in the right graph. (**J**) The capability of EMT and proliferation was impaired with the depletion of CSF3 in OVCA433 cells with mutated NOTCH1. The expression of the related molecule was evaluated by Western blot. * *p* < 0.05, ** *p* < 0.01, *** *p* < 0.005, **** *p* < 0.0001; n.s., not significant; determined by Student’s *t* test (two-tailed) at each time point.

The downstream pathway activated by *NOTCH1*-p.C720fs mutation was analyzed by RNA sequencing. The heatmap is shown in Figure 3A. The KEGG pathway results of differentially expressed genes showed that cytokine–cytokine receptor interaction ranked first (Figure 3B). As a result, CXCL1, CXCL2, CXCL3, CXCL8, CSF3 and IL1B were determined to be candidates regulated by *NOTCH1*-p.C720fs mutation. Then, the expression of NOTCH1 and six candidate genes was verified by RT–PCR (Figure 3C,D). The expression of all candidate genes except CXCL1 in cells with mutated NOTCH1 was more than twice as high as that in cells with wild-type NOTCH1, which was confirmed by Western blotting (Figure 3E). To further identify the downstream effector of *NOTCH1*-p.C720fs mutation, the mRNA and protein expression levels of the five candidates were suppressed with siRNA (Figure 3F,G). Migration was significantly decreased when CXCL2 and CSF3 were knocked down, but not when the other factors were knocked down (Figure 3H). Furthermore, the expression of CXCL2 and CSF3 was knocked down by two siRNAs and confirmed (Figure 3I,J). Migration was significantly inhibited when CSF3, but not CXCL2, was knocked down in both transwell and wound healing assays (Figure 3K,L). The expression patterns of N-cadherin and vimentin were decreased by knocking down CSF3 (Figure 3M). In summary, knocking down CSF3 reversed the enhancement of migration mediated by the *NOTCH1* mutation. Thus, CSF3 is the downstream effector by which *NOTCH1*- p.C720fs mutation influences epithelial-mesenchymal transition.

### 2.4. NOTCH1-p.C720fs Mutation Promotes Implantation and Lymph Node Metastasis of Ovarian Cancer In Vivo, Which Can Be Inhibited by LY3039478

**Figure 4:**
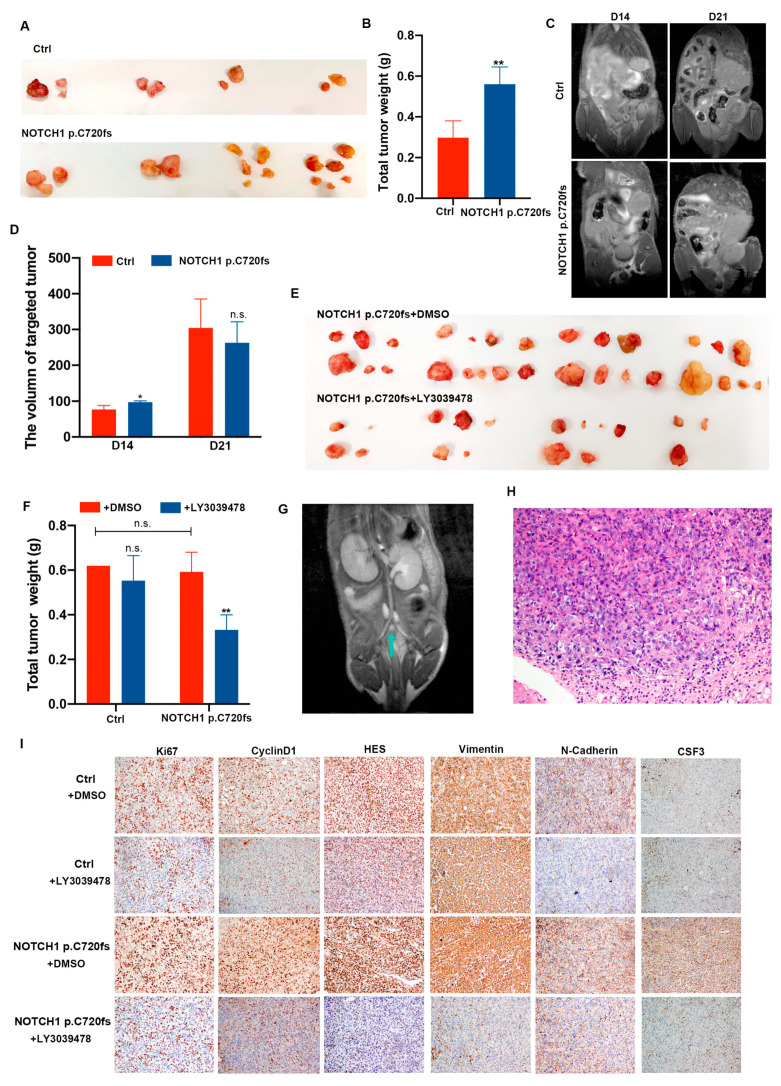
*NOTCH1*-p.C720fs mutation promotes implantation and the lymph node metastasis of ovarian cancer in vivo, which can be inhibited by LY3039478. (**A**,**B**) *NOTCH1*-p.C720fs mutation promotes peritoneal implanting in ovarian cancer. Briefly, 1 × 10^7^ of HeyA8 cells and that with *NOTCH1*-p.C720fs mutation were injected intraperitoneally into the abdomen of four mice. The mice were sacrificed, and all metastatic tumors in the abdomen were dissected on day 16. Data shown are representative images of all metastases (**A**) and total tumor weight of all metastases (**B**). (**C**–**F**) LY3039478 is effective in controlling metastasis in ovarian cancer, which was more remarkable for tumor harboring mutated NOTCH1. Briefly, 7 × 10^6^ of HeyA8 cells and that with mutated NOTCH1 were injected intraperitoneally into the abdomen of eight mice. On day 14, mice in the control and mutant group were examined by MRI and began to take LY3039478 (8 mg/kg), then the selected mice were taken for MRI detection again 7 days later to assess the efficacy of LY3039478. Data shown are representative MRI image (**C**) and quantitative analysis of targeted tumor volume (**D**) of mice before and after the treatment of LY3039478. The mice were sacrificed, and all abdominal tumors were removed on day 21. (**E**) All extracted tumors were presented in mutant group. (**F**) The total tumor weight was calculated and analyzed. (**G**,**H**) *NOTCH1*-p.C720fs mutation promotes the formation of lymph node metastasis in ovarian cancer xenografts. (**G**) The MRI image represented the enlarged retroperitoneal lymph node in mutant group treated with DMSO. (**H**) The HE staining of tumors indicated by arrow in (**G**). (**I**) Four groups of targeted tumors were processed for IHC assay to detect the expression of indicated proteins. Data shown represent the mean ± SEM. * *p* < 0.05, ** *p* < 0.01, n.s., not significant.

To further investigate the role of *NOTCH1*-p.C720fs mutation in ovarian cancer metastasis in vivo, we assessed the effect of *NOTCH1*-p.C720fs mutation on the metastatic ability of xenografts in nude mice. HeyA8 cells (1 × 10^7^) were injected intraperitoneally into the abdomen of four mice. The mice were sacrificed, and the tumors were dissected on day 16. All metastases were removed and photographed, as shown in Figure 4A. The metastases in the mutant group were significantly larger and more abundant than those in the wild-type group, and nearly 2 mL of pale-yellow ascites was found in two mice with *NOTCH1*-p.C720fs mutation, while no mice with ascites were found in the wild-type group. The total weight of metastatic tumors in each mouse of the mutant group was much higher than that of those in the control group (0.3 g vs. 0.56 g, *p* < 0.01, Figure 4B). Given that 1 × 10^7^ cells injected intraperitoneally was too much for the tumor to multiply, it was decreased to 7 × 10^6^ cells in the following study to explore the inhibitory effect of LY3039487. On day 14, three mice in the control and mutant groups were chosen for MRI detection to evaluate the size of the targeted lesion as the size before treatment. The average volumes in the control and mutant groups were 76.8 mm^3^ vs. 97.5 mm^3^ at day 14 (*p* = 0.038). After 7 days of LY3039487 treatment, the sizes of the targeted lesions increased to 304.2 mm^3^ vs. 262.6 mm^3^ in the control and mutant groups, respectively (*p* = 0.51, Figure 4C,D). Resections were performed for all the mice; half of them in the control group did not show any abdominal implant foci, and all tumors in the other half of the tumor-forming mice are shown in Supplementary Figure S2A. All tumors in the mice with NOTCH1-p.C720fs mutation are shown in Figure 4E. Figure 4F displays the total weight of all dissected tumors in the two groups. The total tumor weight of mice with NOTCH1^p.C720fs^ after treatment with LY3039478 was significantly decreased compared with that after treatment with DMSO (0.33 g vs. 0.59 g, *p* < 0.01). Compared with DMSO, LY3039487 inhibited proliferation in tumors with NOTCH1^p.C720fs^. However, there was no positive result regarding the effect of LY3039487 on mice in the control group because half of the mice did not exhibit tumorigenicity, although an inhibitory trend was revealed.

It is worth mentioning that enlarged lymph nodes were found in one mouse of the mutant group treated with DMSO, and this observation was clearly shown by MRI examination of one mouse (Figure 4G); the lymph node indicated by the arrow was removed and subjected to HE staining (Figure 4H). Proliferation and metastasis indicators, such as Ki67, CyclinD1 and vimentin, as well as CSF3, were detected in the tumors of the four groups, demonstrating that the proliferation and metastasis ability of the mutant group treated with DMSO was significantly greater than that of the wild-type group. In addition, LY3039478 inhibited proliferation and metastasis in both the wild-type and mutant groups, but the effect was more obvious in the mice with NOTCH1^p.C720fs^ (Figure 4I).

The side effects of LY3039487 in vivo were briefly evaluated. The mice showed marked emaciation when treated with LY3039487, and their body weight was significantly decreased compared to that of those treated with DMSO (*p* < 0.01, Supplementary Figure S2B). However, the kidney and liver were extracted and subjected to HE staining. There was no evident tissue damage (Supplementary Figure S2C). Thus, it was speculated that the loss of mouse weight was caused by poor appetite.

### 2.5. NOTCH1 Mutations Were more Prominent in Lymph Node Metastases of Ovarian Cancer Than in other Metastatic Sites in the Xenograft Model

**Figure 5:**
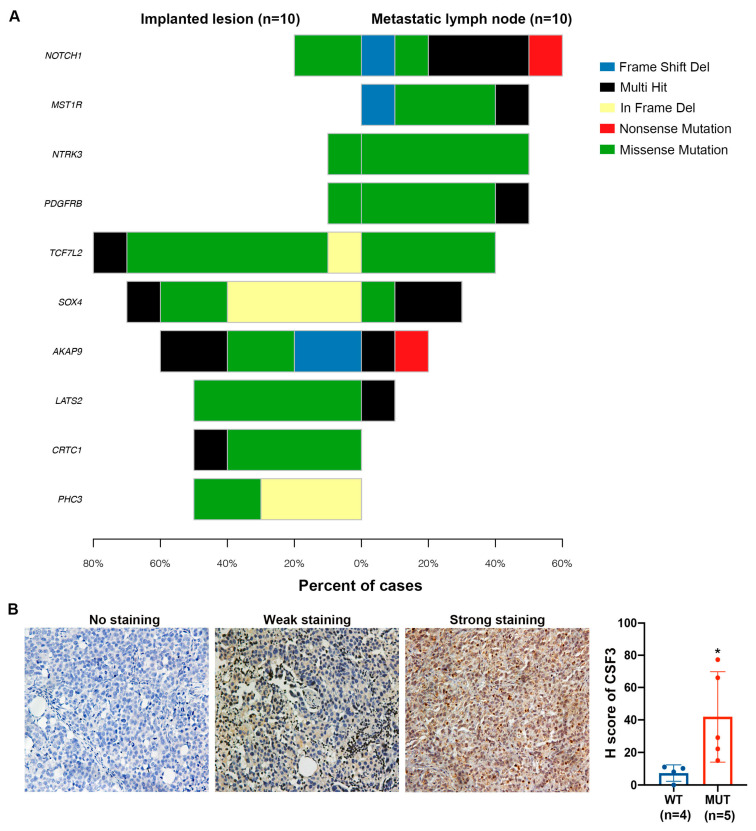
*NOTCH1* mutations are more prominent in metastatic lymph nodes of ovarian cancer than in other implant metastatic sites. (**A**,**B**) Ten paired abdominal implant lesions and metastatic lymph nodes of ovarian cancer were subjected to WES and IHC detection to assess the difference of driver genes and the expression of downstream CSF3 in implant lesions and metastatic lymph nodes. (**A**) The heat map of driver genes in 10 paired abdominal implant lesions and metastatic lymph nodes of ovarian cancer. (**B**) The expression level of CSF3 in lymph nodes harboring mutated NOTCH1 was significantly higher than that with wild-type NOTCH1. The representative image of scoring criteria on the expression of CSF3: “no staining”, “weak staining”, and “strong staining”, with the quantitative analysis of H score shown on the right. * *p* < 0.05.

To further explore whether *NOTCH1* mutation is a driver event of lymph node metastasis in ovarian cancer, WES was performed on 10 paired abdominal implant lesions and metastatic lymph nodes of ovarian cancer. The SNV is shown in Figure 5A. Notably, *NOTCH1* mutations were found in 60% (6/10) of metastatic lymph nodes and 20% (2/10) of implanted lesions. The characteristics of the 10 patients and the mutations are detailed in Table 1. 

Next, the expression of CSF3 in tissue with mutated and wild-type *NOTCH1* was compared by IHC. The H score of CSF3 in metastatic lymph nodes with *NOTCH1* mutation was significantly higher than that in metastatic lymph nodes with NOTCH1^WT^, indicating that CSF3 was a downstream effector of *NOTCH1* mutation (*p* = 0.046, Figure 5B).

## 3. Discussion

Platinum-resistant ovarian cancer has always been difficult to treat. The prognosis is poor, with a reported tumor response rate of 10–15%, median progression-free survival (PFS) of 3 to 4 months, and median overall survival of 9 to 12 months in most phase III trials [8]. Before the administration of olaparib, the donor of FDOVL cells relapsed after 4 months of platinum-based chemotherapy. From this perspective, FDOVL is a platinum-resistant cell line. The establishment of the FDOVL cell line was important to better understand the underlying molecular mechanism of platinum resistance.

The metastasis of advanced ovarian cancer is very common. Pelvic implant metastasis, lymph node metastasis and hematogenous metastasis are the main metastatic pathways. The mechanisms are intricate and involve the tumor microenvironment and cancer stem-like cells [9]. The molecular mechanisms of lymph node metastasis and common implant metastasis are different, but there are few studies on this topic. Understanding the mechanism of lymph node metastasis in ovarian cancer is crucial for the implementation of prophylactic lymphadenectomy for prolonging disease-free survival (DFS) and for improving prognosis. In this study, we found that *NOTCH1* mutation was detected only in FDOVL cells, and the donor left pelvic lymph node from which they were derived, indicating that *NOTCH1* mutation may be one of the driving events for lymph node metastasis in ovarian cancer. *NOTCH1* mutation is most common in lymphocytic leukemia [10,11], esophageal squamous cell carcinoma [12,13], and head and neck squamous cell carcinoma (HNCC) [14,15], and it has been shown to be oncogenic. Notch signaling is also aberrantly activated in breast cancer, non-small cell lung cancer and hematological malignancies, and is involved in many aspects of tumor biology, from angiogenesis to cancer stem cell maintenance to tumor immunity [16]. In addition, its function in EMT was described based on crosstalk between HIF-1α and estrogen with NOTCH signaling in breast cancer [17]. Jackstadt et al. reported that pathological NOTCH1 signaling in colorectal cancer (CRC) cells rewired the tumor microenvironment to drive metastasis [18]. In the present study, *NOTCH1* mutation promoted the migration and invasion of ovarian cancer cells in vitro and in vivo, and these effects could be attenuated by NOTCH1 inhibitor (LY3039478) treatment, especially in cells with *NOTCH1* mutation. LY3039478 displayed preferable clinical activity and was well tolerated for advanced and metastatic cancer in a first-in-human study [19]. Whether it could be effective in ovarian cancer patients with lymph node metastases still needs further study. Although another gamma-secretase inhibitor, RO4929097, showed insufficient activity as a monotherapy for platinum-resistant ovarian cancer, further investigation of the indications of combining a NOTCH inhibitor with another targeted therapy or chemotherapy for ovarian cancer is ongoing. In addition, it was reported that *NOTCH1* mutation exhibits oncogenic properties via the activation of the EGFR-PI3K-AKT pathway in HNCC [14], and HNCC with mutated *NOTCH1* was more vulnerable to therapeutic PI3K/mTOR inhibition [20], which implied that PI3K inhibitors may benefit patients with ovarian cancer lymph node metastasis.

CSF3 was identified as the downstream effector by which *NOTCH1* mutation promotes metastasis in our study. In fact, the regulatory relationship between NOTCH signaling and CSF3 has revealed that activated NOTCH could affect the promoter recruitment of CSF3, which functions in barrier immunity [21]. CSF3 is a member of the CSF superfamily and is considered as a growth factor with the ability to stimulate the proliferation and differentiation of cancer cells [22]. It also has been shown to regulate the innate and adaptive immune systems [23,24]. Activated CSF3 is associated with decreased overall survival (OS) and is often accompanied by inflammation and signal transduction pathways [25]. In colorectal and pancreatic cancer, the expression of CSF3 is elevated and it increases tumor cell proliferation and migration and shows high immunosuppressive activity [26,27]. Nevertheless, CSF3 is strongly upregulated in a breast cancer cell line with high potential for lymph node metastasis [28]. Thus, the function of CSF3 in lymph node metastasis in ovarian cancer still needs further investigation, and CSF3 may be a promising target for the treatment of metastatic lymph nodes.

In a variety of solid tumors, it was revealed that the genome evolved during metastasis and relapse. Some driver mutations were acquired during distant metastases and were not seen in the primary tumor [29,30]. In our study, *NAV3*, *KIAA1109* and *PRDM2* mutations, especially *BRCA1* missense mutations, were detected in all samples of recurrent tumors, but were excluded from the first debulking surgery. It was reported that approximately 45% of patients with platinum-resistant recurrent ovarian cancer have secondary mutations restoring BRCA1/2; this rate is significantly higher than that in platinum-sensitive recurrent cancer, and the secondary somatic mutations that restore *BRCA1/2* in carcinomas with germline *BRCA1/2* mutations predict resistance to platinum chemotherapy or PARPi [31]. In this case, the patient still relapsed four months after secondary surgery and albumin-bound paclitaxel and cisplatin-based chemotherapy, which was probably due to the secondary mutation in *BRCA1*. Alternatively, the ovarian cancer itself in this patient was platinum resistant. However, the patient achieved a complete response (CR) that lasted for nearly 3 years after oral olaparib administration, probably due to the positive homologous recombination deficiency (HRD) status, which is slightly contradictory to the above claim. Supplementary Figure S3 shows the changes in CA125 from the time she visited our hospital (April 2017) to the most recent evaluation. This case makes us ponder the following two questions: first, is chemotherapy necessary for patients with satisfactory tumor reduction achieved by surgery? Second, is maintenance therapy also suitable for patients with platinum-resistant recurrent ovarian cancer? *BRCA1/2* mutation or HRD positivity may also be molecular markers of maintenance therapy for platinum-resistant recurrent ovarian cancer.

The sequencing results, especially the CNV results, showed that the tumors from similar areas, such as the intrapelvic and left pelvic lymph nodes, displayed similar molecular biological characteristics. In other words, tumors undergo different molecular events during invasion and metastasis to distant sites. Of course, in this regard, the occurrence of lymph node metastasis in ovarian cancer may not have a chronological sequence with the occurrence of epigastric metastasis. Moreover, the occurrence of epigastric metastasis may be due to the gravity factor of tumor implantation and metastasis, but it is unclear whether metastases in different sites could survive and develop due to the molecular features that evolve in more suitable sites.

In summary, these findings provide a new idea for clinical trials of NOTCH inhibitors alone and in combination with other targeted therapies or chemotherapy in the treatment of ovarian cancer with lymph node metastasis.

## 4. Materials and Methods

### 4.1. Medical History

A 53-year-old woman was admitted to Fudan University Shanghai Cancer Center due to ovarian cancer that recurred with retroperitoneal lymph node metastases in August 2017, 4 months after six cycles of PC regimen (paclitaxel +carboplatin) chemotherapy administered at a different hospital (Supplementary Figure S1A). In October 2016, the patient was diagnosed with HGSOC (FIGO IIIC stage) at another hospital and underwent debulking surgery, including pelvic lymphadenectomy. Postoperative pathology indicated that many lymph nodes, such as the para-aortic and left parametrium, were affected. After secondary debulking surgery, the patient was treated with six cycles of albumin-bound paclitaxel and cisplatin but the tumor still recurred with bilateral iliac paravascular lymph node metastases after 4 months of chemotherapy (Supplementary Figure S1B). She underwent a third cytoreductive surgery and then began to take olaparib orally until May 2021. Supplementary Figure S1C shows the site of recurrence. Then, she underwent a fourth debulking surgery, followed by chemotherapy and radiation. In summary, the patient had platinum-resistant recurrent ovarian cancer that recurred mainly in the retroperitoneal lymph nodes.

### 4.2. Establishment of Cell Lines

The patient gave written informed consent before surgery according to institutional guidelines in our cancer center (FUSCC 050432-4-1212B). One of the left pelvic lymph nodes was removed during debulking surgery and sent for primary culture; the cells were stably passaged and named FDOVL [32]. FDOVL cells were cultured in RPMI 1640 medium with 12% fetal bovine serum, 100 IU/mL penicillin, 50 μg/mL streptomycin, 1% sodium pyruvate, 1% HEPES buffer and 1% nonessential amino acids. In addition, the cells were sent to the China General Microbiological Culture Collection Center for preservation (No. 15484) in March 2018.

### 4.3. Growth Characteristics

Next, 2 × 10^5^ FDOVL cells were seeded in 6-well plates. The cells in one well were digested and counted in triplicate every two days. The culture medium in the other wells was routinely exchanged. After five consecutive measurements, the cell proliferation curve was obtained for 10 days and the cell doubling time was calculated.

### 4.4. Chromosome Analysis

Chromosome analysis was performed in FDOVL cells, as described in a previous study [8].

### 4.5. Heterotransplantation of FDOVL Cells

All animal experiments were approved by the Institutional Animal Care and Use Committee of Fudan University and performed according to institutional guidelines. A total of 1 × 10^7^ FDOVL cells (32 generations) were injected subcutaneously into the dorsal flanks of three nude mice (4 weeks, female). The tumor size was measured every 4 days and calculated by longest diameter × shortest diameter^2^ × 0.5. Magnetic resonance imaging (MRI) of transplanted tumors was performed 10 days after the tumor was visible to the naked eye.

### 4.6. Histological Analysis and Immunohistochemistry

The transplanted tumors were surgically removed until the tumor volume reached 100 mm^3^, and then fixed in 10% formalin. Then, the tumors derived from the donor’s pelvic lymph node and the transplanted tumors were subjected to routine hematoxylin and eosin (HE) staining and staining for other markers, including P16 (1:50, ab51243, Abcam, Cambridge, UK), P53 (1:2000, ab32389, Abcam), ER (1:800, 21244-1-AP, Proteintech), PR (1:200, 25871-1-AP, Proteintech, Chicago, IL, USA), PAX8 (1:200, 10336-1-AP, Proteintech), and WT1 (1:500, ab89901, Abcam). Microscopic slides were reviewed by a senior gynecology-dedicated pathologist (Prof. Yang).

Similarly, immunohistochemistry (IHC) was conducted to assess the expression of Ki67 (1:200, AF0198, Affinity, San Francisco, CA, USA), CyclinD1 (1:250, ab134175, Abcam), Hes1 (1:200, ab108937, Abcam), N-cadherin (1:200, #13116, Cell Signaling Technology, Danvers, MA, USA), vimentin (1:200, #5741, Cell Signaling Technology) and CSF3 (1:200, 17185-1-AP, Proteintech) in the tumors extracted from mice. The kidney and liver were also removed for HE staining to evaluate the toxicity of viscera.

Finally, the expression of CSF3 in the corresponding lymph nodes samples from 10 ovarian cancer patients was evaluated by IHC. The scoring criteria for staining intensity were described in our previous research [33].

### 4.7. Whole-Exome Sequencing (WES) and Sanger Sequencing

WES was performed on 10 patients using DNA from both tumor samples and matched noncancerous tissues. The operation was performed according to Illumina’s standard procedure (effective tumor depth of 300×, blood sample of 100×). First, DNA was extracted using the QIAamp DNA FFPE Tissue Kit Print (Qiagen, Hilden, Germany) according to the manufacturer’s protocol. A Paired-end DNA library was prepared according to the manufacturer’s instructions (Agilent, Santa Clara, CA, USA). Samples with a total amount greater than 0.6 µg were used for library preparation. Genomic DNA was fragmented by Covaris sonication to a length of 200–300 bp. The ends of DNA fragments were repaired, and Illumina Adaptor was added (Fast Library Prep Kit, iGeneTech, Beijing, China). The DNA fragments were end-polished, A-tailed and ligated with the full-length adapter. After the sequencing library was constructed, whole exomes were captured with the AIExomeV2 (T192V1T) Enrichment Kit (iGeneTech, Beijing, China) and sequenced on an Illumina platform (Illumina, San Diego, CA, USA) with 150 bp paired end reads. Raw reads were filtered to remove low quality reads using Fast QC. Clean reads were mapped to the reference genome GRCh37 using BWA. After removing duplications, single nucleotide variants (SNVs) and insertions and deletions (indels) were called and annotated using the Genome Analysis Toolkit (GATK) based on dbSNP build 150. All variants were annotated with ANNOVAR. Similarly, the genomic information of FDOVL cells and the corresponding lymph node tissue, as well as the primary focality of the first and second debulking surgeries, were compared by WES. The sequencing coverage and quality statistics for each sample are summarized in Supplementary Table S1.

*BRCA1* and *NOTCH1* mutations in cells were confirmed by Sanger-sequencing, as described in a previous study [32].

### 4.8. Cell Culture and Viral Transfection

The cell lines HEY A8 (RRID: CVCL_8878), OVCA433 (RRID: CVCL_0475), OVCA420 (RRID:CVCL_3935), SK-OV-3 (RRID:CVCL_0532), A2780 (RRID:CVCL_0134), OVCAR-8 (RRID:CVCL_1629), IGROV-1 (RRID:CVCL_1304) and ES-2 (RRID:CVCL_3509) were obtained from the Chinese Academy of Sciences Cell Bank (Shanghai, China), whose cell lines were originally obtained from the American Type Culture Collection. All human cell lines were authenticated using short tandem repeat profiling within the last three years. All experiments were performed with mycoplasma-free cells. Both cell lines were cultured in RPMI 1640 supplemented with 10% fetal bovine serum (FBS), 100 U/mL penicillin and 100 mg/mL streptomycin and incubated at 37 ℃ with 5% CO_2_.

The mutated NOTCH1 plasmid and virus were purchased from Hanyin Biotechnology Limited Company (Shanghai, China). The sequence was displayed in the Supplementary file. The plasmid was subcloned into the lentivirus vector CMV-MCS-3XFLAG-PGK-PURO to construct a recombinant plasmid. Transfection was performed according to a previous study [33]. The establishment of cells with NOTCH1 ^p.C720fs^ was verified by the expression of the Flag tag.

### 4.9. Western Blot Assay

Western blotting was used to evaluate the expression level in the cells, as previously described [33]. Antibodies against hairy and enhancer of split-1 (Hes1), Cyclin D1 (CCND1), E-cadherin and N-cadherin were purchased from Proteintech, Abcam and Cell Signaling Technology. All antibodies were diluted 1:1000.

### 4.10. Transwell and Wound Healing Assays

A total of 2 × 10^5^ cells in 200 µL DMEM without fetal bovine serum were placed in the upper chamber of a Transwell insert (24 wells, 8 µm pore size; Corning Costar) coated with Matrigel (Corning, 356,237). The lower chambers were loaded with NOTCH1 inhibitor in 500 µL DMEM with 10% fetal bovine serum or culture medium only as a control. After incubation at 37 °C for 24 h, the cells were removed from the upper chamber using a cotton swab and the cells on the lower membrane surface were fixed with 4% formalin, stained with 0.1% crystal violet for visualization, and counted under a microscope (Leica).

The cells were seeded at a density of 3 × 10^6^ cells per well into a 6-well plate for the wound healing assay. They were pretreated with or without NOTCH1 inhibitor and then a line was scratched across the center of the well after the cells adhered to the well. After 16 or 24 h, the cells were washed three times with PBS and then quantified using an inverted fluorescence microscope. The migration rate was calculated as follows: [(At = 0 h-At = Δh)/At = 0 h] × 100%, where “At = 0 h” is the area of the wound measured immediately after scratching and “At = Δh” is the area of the wound at the time point 24 h after scratching.

### 4.11. RNA Extraction and Quantitative Real-Time PCR (qRT–PCR)

Total RNA was extracted with TRIzol, and qRT–PCR was performed using the HiScript II One Step qRT–PCR SYBR GREEN Kit (Vazyme Biotech Co., Ltd., Nanjing, Jiangsu, China) in accordance with the manufacturer’s manual. Relative amounts of mRNA were calculated using the comparative method, with β-actin as a housekeeping gene. The results were analyzed with SDS v2.1 software and the 2^−ΔΔ*C*t^ method. The primer sequences are listed in Supplementary Table S2.

### 4.12. RNA Sequencing

Total RNA samples (1 μg) of HeyA8 and OVCA433 cells and those with NOTCH1^p.C720fs^ were treated with VAHTS mRNA Capture Beads (Vazyme, Nanjing, China) to enrich polyA+ RNA. RNA-seq libraries were prepared using the VAHTS mRNA-seq v2 Library Prep Kit for Illumina (Vazyme, Nanjing, China) following the manufacturer’s instructions. Briefly, polyA+ RNA samples (approximately 100 ng) were fragmented and then used for first- and second-strand cDNA synthesis with random hexamer primers. The cDNA fragments were treated with the DNA End Repair Kit to repair the ends, modified with Klenow to add an A at the 3′ end of the DNA fragments, and finally ligated to adapters. Purified dsDNA was subjected to 12 cycles of PCR amplification, and the libraries were sequenced by Illumina sequencing platform (NovaSeq 6000) on a 150 bp paired-end run. Sequencing reads from RNA-seq data were aligned with the reference human genome (Genome Reference Consortium GRCh38) using the spliced read aligner HISAT2 in Ensemble. Gene expression levels (read counts) and FPKM (fragments per kilobase of transcript per million mapped reads) values were calculated using featureCount. The Database for Annotation, Visualization, and Integrated Discovery (DAVID) was employed for explore GO and KEGG enrichment analysis of the differentially expressed genes.

### 4.13. Small Interfering RNA (siRNA) Transfection

SiRNAs against CXCL2, CXCL3, CXCL8, CSF3 and IL1B, which were obtained from RuiBo Biotechnology Limited Company (Guang Zhou, China), were transfected into OVCA433 cells with *NOTCH1*-p.C720fs mutation with FuGENE HD transfection reagent (Promega). The sequences are shown in Supplementary Table S3.

### 4.14. Patients and Specimens

The present study was approved by the Ethics Committee at Fudan University Shanghai Cancer Center (1908205-18) and conducted in accordance with the approved guidelines. Ten patients with ovarian cancer were recruited from June 2020 to December 2020. The specimens, including the peritoneal implants and metastatic lymph nodes, were collected during the procedures regardless of primary or secondary debulking surgery and stored in RNA later at −80 ℃. All lymph nodes subjected to RNA sequencing were confirmed to have tumor infiltration by HE staining, with a tumor invasion area greater than 50%. Informed consent was obtained from each patient prior to treatment.

### 4.15. Establishment and Treatment of Xenografts

A total of 1 × 10^7^ cells (OVCA433 and OVCA433 with *NOTCH1* mutation) mixed with 100 µL of phosphate-buffered saline (PBS) were injected intraperitoneally into BALB/c-nu mice (female, 4 weeks of age; Shanghai SLAC Laboratory Animal Co. Ltd., Shanghai, China). Each group had four mice to compare the tumorigenicity of OVCA433 with NOTCH1^WT^ and *NOTCH1*^p.C720fs^. The mice were sacrificed on the 16th day. All implants in the abdominal cavity were removed, weighed, and photographed. To further evaluate the efficiency of the NOTCH inhibitor, 7 × 10^6^ of HeyA8 cells with and without *NOTCH1*-p.C720fs mixed with 100 µL of PBS were injected intraperitoneally into eight mice. The mice were divided into two groups and treated as follows: (1) DMSO and (2) LY3039478 (8 mg/kg). MRI examination was performed before administration and execution to assess the efficiency. The tumor volume was calculated as the longest diameter × the shortest diameter^2^ × 0.5. The mice were raised until their general state worsened. Similarly, all implants, including the mesenteric lesions and suspicious enlarged lymph nodes, were removed and weighed. The largest lesion was fixed and embedded for IHC analysis, including Ki67, Cyclin D1, Hes1, Vimentin, N-cadherin and CSF3. Parts of the liver and kidney were also extracted for HE staining to evaluate drug toxicity.

## Figures and Tables

**Table 1:** The characteristics and mutation information of the 10 paired ovarian cancer patients.

Sample Number	Sample Number	Source of Tumor	*NOTCH1* Mutation	Mutation Type	*NOTCH1* Mutation	Mutation Type
1	JS-3	Primary	-		NM_017617:exon25:c.C4056A:p.C1352X	SNP (stopgain)
2	JS-10	Primary	-		(1) NM_017617:exon34:c.A6325G:p.I2109V (2) NM_017617:exon12:c.G1993A:p.A665T	SNP (nonsynonymous)
3	JS-12	Primary	NM_017617:exon11:c.C1860G:p.D620E	SNP (nonsynonymous)	(1) NM_017617:exon34:c.A6317C:p.H2106P (2) NM_017617:exon26:c.T4706C:p.L1569P (3) NM_017617:exon11:c.C1860G:p.D620E	SNP (nonsynonymous)
4	JS-13	Primary	-		NM_017617:exon34:c.6392delG:p.G2131fs	Indel (frameshift deletion)
5	JS-14	Recurrent	-		(1) NM_017617:exon26:c.T4706C:p.L1569P (2) NM_017617:exon30:c.C5521G:p.R1841G	SNP (nonsynonymous)
6	JS-18	Primary	-		-	
7	JS-22	Primary	NM_017617:exon34:c.A6317C:p.H2106P	SNP (nonsynonymous)	-	
8	JS-25	Primary	-		-	
9	JS-26	Primary	-		-	
10	JS-33	Primary	-		NM_017617:exon4:c.C498G:p.C166W	SNP (nonsynonymous)

## Data Availability

The raw WES and RNA-seq data generated in this study are available in the Genome Sequence Archive (GSA) database. Other data that support the findings of this study are available from the corresponding author upon request.

## Supplementary Materials

The following supporting information can be downloaded at: https://www.mdpi.com/article/10.3390/ijms24065091/s1.
