# Supplementary material for: A NOTCH1 Mutation Found in a Newly Established Ovarian Cancer Cell Line (FDOVL) Promotes Lymph Node Metastasis in Ovarian Cancer
Source: Int J Mol Sci. 2023 Mar 7;24(6):5091. doi: 10.3390/ijms24065091 (PMC10049685; doi:10.3390/ijms24065091)
Supplement: Supplementary file 1 [file ijms-24-05091-s001.zip › figures.pdf]

Supplementary Figure S1. The relapsed PET-CT image of the donor of FDOVL cells, the arrow indicated the major recurrent foci. (*A*) The image photographed at first relapse; FDOVL cells were derived from the tumor indicated by the arrow on the left. (*B*) The image photographed at second relapse. (*C*) The image photographed at third relapse.

Supplementary Figure S2. LY3039478 leads to weight loss in mice but without significant hepatorenal toxicity. The mice with wild-type NOTCH1 were treated with LY3039478. The tumor image was shown in (*A*). The mice with wild-type and mutated NOTCH1 were treated with LY3038479, and the weight was evaluated and analyzed in (*B*). No obvious hepatorenal toxicity was shown after the treatment of LY3039478. The kidneys and livers were extracted and subjected to HE stains to assess the toxicity of viscera. Representative image was presented in (*C*).

Supplementary Figure S3. The curve of CA125 in FDOVL cell line donor patient during the whole treatment process. The key treatment points were indicated by arrow.

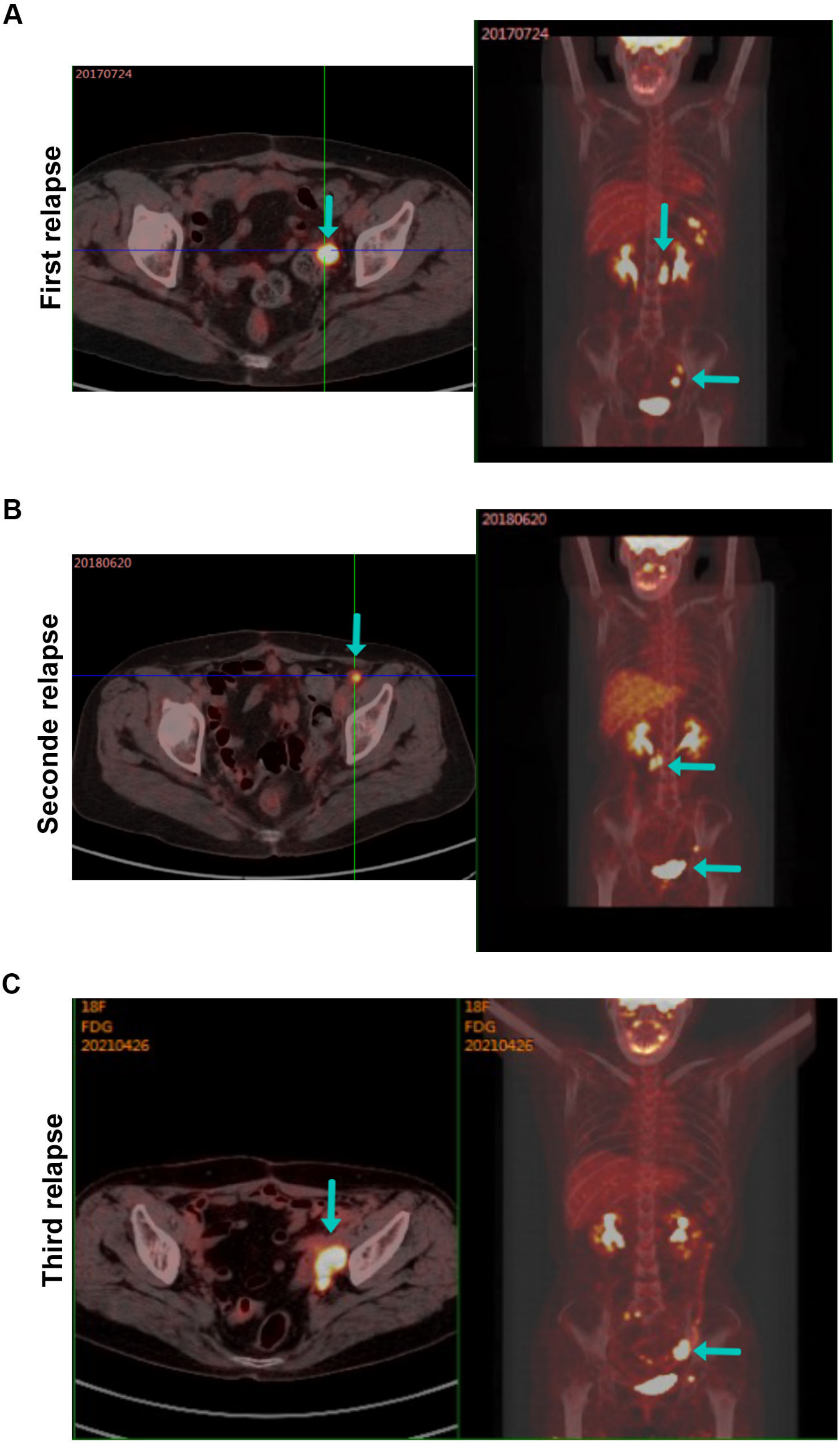
A
First relapse
20170724
20170724
B
Seconde relapse
20180620
20180620
C
Third relapse
18F
FDG
20210426
18F
FDG
20210426
Fig.S1

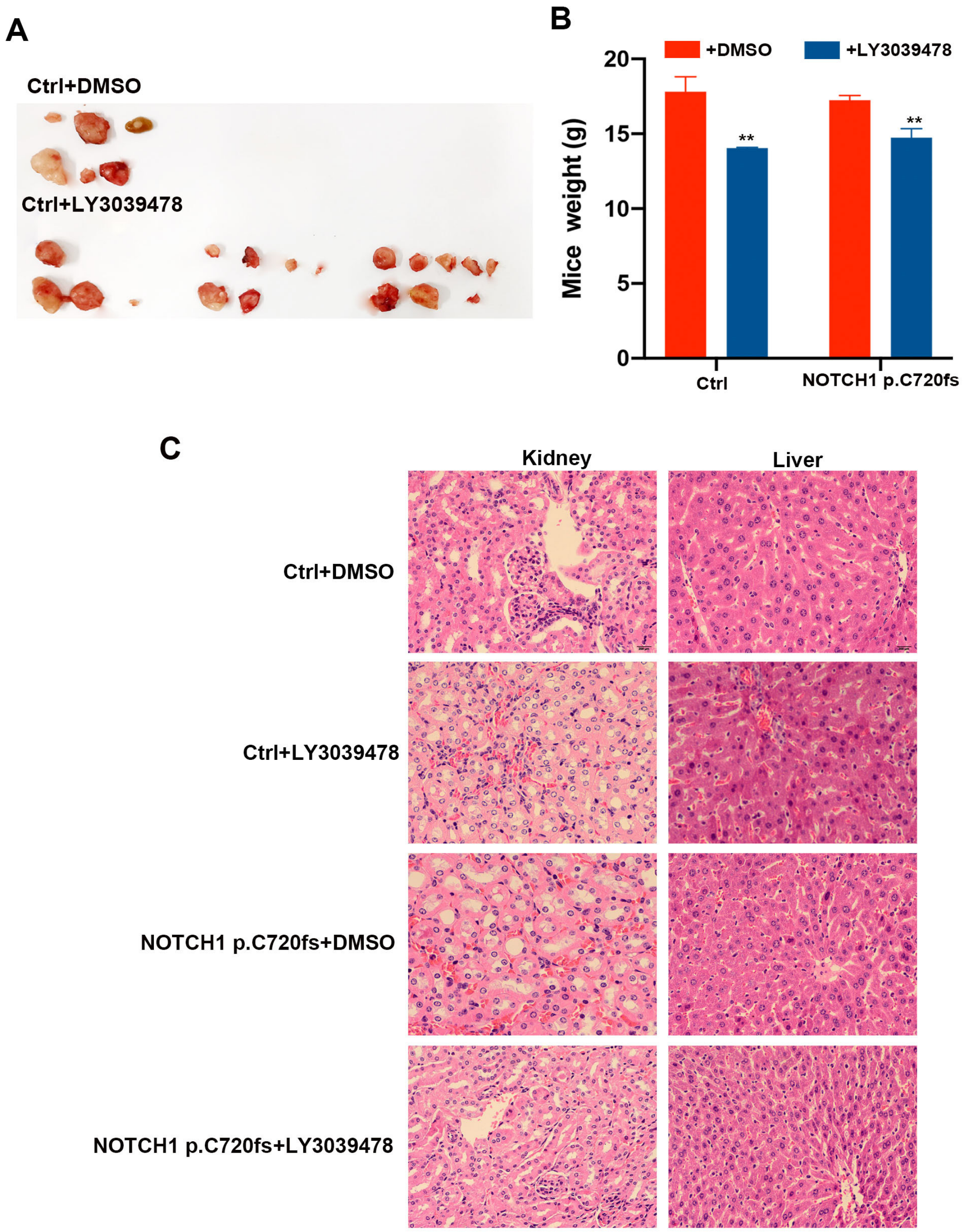
A
Ctrl+DMSO
Ctrl+LY3039478
B
+DMSO
+LY3039478
20
15
10
5
0
Mice weight (g)
**
**
Ctrl
NOTCH1 p.C720fs
C
Kidney
Liver
Ctrl+DMSO
Ctrl+LY3039478
NOTCH1 p.C720fs+DMSO
NOTCH1 p.C720fs+LY3039478

Fig.S2

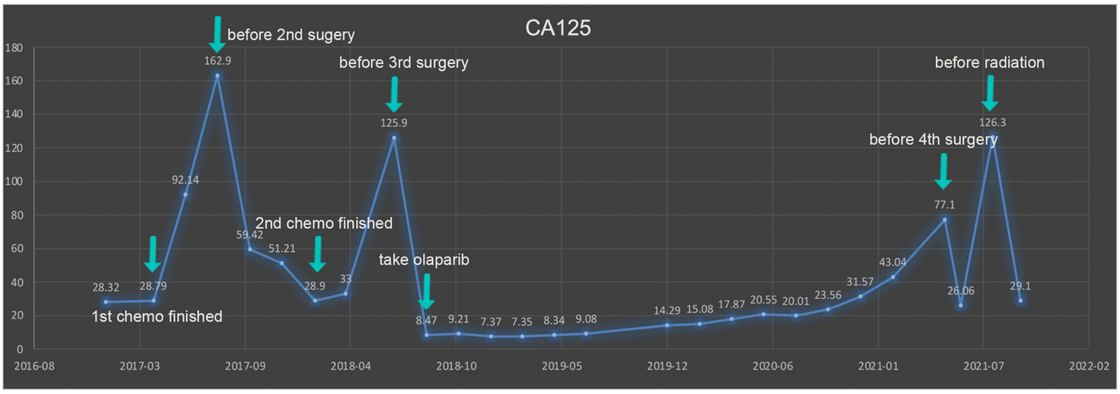
CA125
before 2nd sugery
before 3rd surgery
before radiation
before 4th surgery
2nd chemo finished
take olaparib
1st chemo finished
162.9
92.14
59.42
51.21
28.32
28.79
28.9
33
125.9
8.47
9.21
7.37
7.35
8.34
9.08
14.29
15.08
17.87
20.55
20.01
23.56
31.57
43.04
77.1
26.06
126.3
29.1
180
160
140
120
100
80
60
40
20
0
2016-08
2017-03
2017-09
2018-04
2018-10
2019-05
2019-12
2020-06
2021-01
2021-07
2022-02

Figure S3
