# Supplementary material for: A NOTCH1 Mutation Found in a Newly Established Ovarian Cancer Cell Line (FDOVL) Promotes Lymph Node Metastasis in Ovarian Cancer
Source: Int J Mol Sci. 2023 Mar 7;24(6):5091. doi: 10.3390/ijms24065091 (PMC10049685; doi:10.3390/ijms24065091)
Supplement: Supplementary file 1 [file ijms-24-05091-s001.zip › supple-file.pdf]

Supplementary file: The sequence of NOTCH1 p.C720fs mutation

ATGCCGCCGCTCCTGGCGCCCCTGCTCTGCCTGGCGCTGCTGCCCGCGCTCGCCGCAC

GAGGCCCGCGATGCTCCCAGCCCGGTGAGACCTGCCTGAATGGCGGGAAGTGTGAAG

CGGCCAATGGCACGGAGGCCTGCGTCTGTGGCGGGGCCTTCGTGGGCCCGCGATGCC

AGGACCCCAACCCGTGCCTCAGCACCCCCTGCAAGAACGCCGGGACATGCCACGTGG

TGGACCGCAGAGGCGTGGCAGACTATGCCTGCAGCTGTGCCCTGGGCTTCTCTGGGC

CCCTCTGCCTGACACCCCTGGACAATGCCTGCCTCACCAACCCCTGCCGCAACGGGGG

CACCTGCGACCTGCTCACGCTGACGGAGTACAAGTGCCGCTGCCCGCCCGGCTGGTC

AGGGAAATCGTGCCAGCAGGCTGACCCGTGCGCCTCCAACCCCTGCGCCAACGGTGG

CCAGTGCCTGCCCTTCGAGGCCTCCTACATCTGCCACTGCCCACCCAGCTTCCATGGC

CCCACCTGCCGGCAGGATGTCAACGAGTGTGGCCAGAAGCCCGGGCTTTGCCGCCAC

GGAGGCACCTGCCACAACGAGGTCGGCTCCTACCGCTGCGTCTGCCGCGCCACCCAC

ACTGGCCCCAACTGCGAGCGGCCCTACGTGCCCTGCAGCCCCTCGCCCTGCCAGAAC

GGGGGCACCTGCCGCCCCACGGGCGACGTCACCCACGAGTGTGCCTGCCTGCCAGGC

TTCACCGGCCAGAACTGTGAGGAAAATATCGACGATTGTCCAGGAAACAACTGCAAG

AACGGGGGTGCCTGTGTGGACGGCGTGAACACCTACAACTGCCGCTGCCCGCCAGAG

TGGACAGGTCAGTACTGTACCGAGGATGTGGACGAGTGCCAGCTGATGCCAAATGCC

TGCCAGAACGGCGGGACCTGCCACAACACCCACGGTGGCTACAACTGCGTGTGTGTC

AACGGCTGGACTGGTGAGGACTGCAGCGAGAACATTGATGACTGTGCCAGCGCCGCC

TGCTTCCACGGCGCCACCTGCCATGACCGTGTGGCCTCCTTCTACTGCGAGTGTCCCC

ATGGCCGCACAGGTCTGCTGTGCCACCTCAACGACGCATGCATCAGCAACCCCTGTA

ACGAGGGCTCCAACTGCGACACCAACCCTGTCAATGGCAAGGCCATCTGCACCTGCC

CCTCGGGGTACACGGGCCCGGCCTGCAGCCAGGACGTGGATGAGTGCTCGCTGGGTG

CCAACCCCTGCGAGCATGCGGGCAAGTGCATCAACACGCTGGGCTCCTTCGAGTGCC

AGTGTCTGCAGGGCTACACGGGCCCCCGATGCGAGATCGACGTCAACGAGTGCGTCT

CGAACCCGTGCCAGAACGACGCCACCTGCCTGGACCAGATTGGGGAGTTCCAGTGCA

TCTGCATGCCCGGCTACGAGGGTGTGCACTGCGAGGTCAACACAGACGAGTGTGCCA

GCAGCCCCTGCCTGCACAATGGCCGCTGCCTGGACAAGATCAATGAGTTCCAGTGCG

AGTGCCCCACGGGCTTCACTGGGCATCTGTGCCAGTACGATGTGGACGAGTGTGCCA

GCACCCCCTGCAAGAATGGTGCCAAGTGCCTGGACGGACCCAACACTTACACCTGTG

TGTGCACGGAAGGGTACACGGGGACGCACTGCGAGGTGGACATCGATGAGTGCGAC

CCCGACCCCTGCCACTACGGCTCCTGCAAGGACGGCGTCGCCACCTTCACCTGCCTCT

GCCGCCCAGGCTACACGGGCCACCACTGCGAGACCAACATCAACGAGTGCTCCAGCC

AGCCCTGCCGCCACGGGGGCACCTGCCAGGACCGCGACAACGCCTACCTCTGCTTCT

GCCTGAAGGGGACCACAGGACCCAACTGCGAGATCAACCTGGATGACTGTGCCAGCA

GCCCCTGCGACTCGGGCACCTGTCTGGACAAGATCGATGGCTACGAGTGTGCCTGTG

AGCCGGGCTACACAGGGAGCATGTGTAACATCAACATCGATGAGTGTGCGGGCAACC

CCTGCCACAACGGGGGCACCTGCGAGGACGGCATCAATGGCTTCACCCCCCGAGGGC

TACCACGACCCCACCTGCCTGTCTGA
