# Supplementary material for: A NOTCH1 Mutation Found in a Newly Established Ovarian Cancer Cell Line (FDOVL) Promotes Lymph Node Metastasis in Ovarian Cancer
Source: Int J Mol Sci. 2023 Mar 7;24(6):5091. doi: 10.3390/ijms24065091 (PMC10049685; doi:10.3390/ijms24065091)
Supplement: Supplementary file 1 [file ijms-24-05091-s001.zip › Supplementary table.pdf]

**Supplementary Table S1. The sequencing coverage and quality statistics of WES.**

| Sample ID | Total number of sequenced reads | Total number of uniquely mapped non-duplicate reads | Total number of covered bases | Median coverage (and range) per base | Percentage of targeted bases with coverage ≥10 |
|---|---|---|---|---|---|
| JS-3 pri | 12403.71 | 65.29 | 39876406 | 130.09 | 99.43 |
| JS-3LN | 12776.12 | 66.38 | 39874427 | 121.11 | 99.35 |
| JS-10 pri | 12128.88 | 63.39 | 39873531 | 117.86 | 99.24 |
| JS-10LN | 12794.73 | 67.45 | 39874258 | 117.8 | 99.22 |
| JS-12pri | 13234.95 | 69.84 | 39873315 | 127.2 | 99.31 |
| JS-12LN | 9571.61 | 54.1 | 39839550 | 104.02 | 98.81 |
| JS-13pri | 12718.37 | 66.41 | 39872733 | 122.3 | 99.3 |
| JS-13LN | 12933.06 | 68.12 | 39873923 | 127.41 | 99.4 |
| JS-14 pri | 14900.81 | 77.25 | 39888546 | 146.89 | 99.22 |
| JS-14 LN | 10717.84 | 57.77 | 39869808 | 94.5 | 98.94 |
| JS-18 pri | 10361.75 | 52.76 | 39863583 | 95.36 | 98.97 |
| JS-18 LN | 13471.51 | 71.08 | 39878019 | 131.88 | 99.41 |
| JS-22 pri | 11973.18 | 61.76 | 39870791 | 113.78 | 99.25 |
| JS-22 LN | 10993.45 | 58.43 | 39869724 | 107.41 | 99.21 |
| JS-25 pri | 11980.7 | 62.79 | 39872976 | 115.39 | 99.18 |
| JS-25 LN | 11336.6 | 60.48 | 39869373 | 111.23 | 98.95 |
| JS-26 pri | 12341.66 | 65.62 | 39870719 | 120.74 | 99.16 |
| JS-26 LN | 11798.44 | 63.24 | 39875727 | 116.72 | 99.32 |
| JS-33 pri | 12120.44 | 63.6 | 39875646 | 119.55 | 99.34 |
| JS-33 LN | 13464.06 | 70.53 | 39880472 | 127.89 | 99.37 |

**Supplementary Table S2. The primer sequences of Qpcr.**

| Gene Name | Forward Primer | Reverse Primer |
|---|---|---|
| **NOTCH1** | CGCTGACGGAGTACAAGTG | GTAGGAGCCGACCTCGTTG |
| **CXCL1** | GCGGGCTGCATCAGTGGA | CGGGACTTACATGACTTCGGT |
| **CXCL2** | GCTTGTCTCAACCCCGCATC | TGGATTTGCCATTTTTCAGCATCTT |
| **CXCL3** | CCAAACCGAAGTCATAGCCAC | TGCTCCCCTTGTTCAGTATCT |
| **CXCL8** | TTTTGCCAAGGAGTGCTAAAGA | AACCCTCTGCACCCAGTTTTC |
| **CSF3** | GCTGCTTGAGCCAACTCCATA | GAACGCGGTACGACACCTC |
| **IL1B** | AGCTACGAATCTCCGACCAC | CGTTATCCCATGTGTCGAAGAA |

**Supplementary Table S3. The sequence of SiRNA.**

| Gene Name | The Sequence |
|---|---|
| **CXCL2** | GGGCAGAAAGCTTGTCTCA |
| | ACATCCAAAGTGTGAAGGT |
| **CSF3** | GCTCAAGTGCTTAGAGCAA |
| | CTGCTTGAGCCAACTCCAT |
| **CXCL3** | CATCCAAAGTGTGAATGTA |
| **CXCL8** | CTTAGATGTCAGTGCATAA |
| **IL1B** | CGATGCACCTGTACGATCA |

**Supplementary Table S4. Differentially expressed CNV driver genes in the five samples.**

| Gene_Symbol | Locus_ID | Cytoband | First primary tumor | FDOVL | Donor (left pelvic lymph nodes) | Pelvic tumor | Upper abdominal tumor |
|---|---|---|---|---|---|---|---|
| **SOX2** | 6657 | 3q26.33 | 1 | 2 | 2 | 2 | 2 |
| **PIK3CA** | 5290 | 3q26.32 | 1 | 2 | 2 | 2 | 2 |
| **RAF1** | 5894 | 3p25.2 | 0 | 2 | 1 | 2 | 2 |
| **MAPK1** | 5594 | 22q11.21 | 0 | 1 | 1 | 2 | 1 |
| **APC** | 324 | 5q22.2 | 0 | -1 | -1 | -1 | -1 |
| **ARID1A** | 8289 | 1p36.11 | 0 | -1 | -1 | -1 | -1 |
| **AXIN1** | 8312 | 16p13.3 | 0 | -1 | -1 | -1 | -1 |
| **BAP1** | 8314 | 3p21.1 | 0 | -1 | -1 | -1 | -1 |
| **BAZ1A** | 11177 | 14q13.1 | 0 | -1 | -1 | -1 | -1 |
| **EML4** | 27436 | 2p21 | 0 | -1 | -1 | -1 | -1 |
| **GRIN2A** | 2903 | 16p13.2 | 0 | -1 | -1 | -1 | -1 |
| **KLF4** | 9314 | 9q31.2 | 0 | -1 | -1 | -1 | -1 |
| **MAX** | 4149 | 14q23.3 | 0 | -1 | -1 | -1 | -1 |
| **MSH2** | 4436 | 2p21 | 0 | -1 | -1 | -1 | -1 |
| **MSH6** | 2956 | 2p16.3 | 0 | -1 | -1 | -1 | -1 |
| **PBRM1** | 55193 | 3p21.1 | 0 | -1 | -1 | -1 | -1 |

| | | | | | | | |
|---|---|---|---|---|---|---|---|
| **PTCH1** | 5727 | 9q22.32 | 0 | -1 | -1 | -1 | -1 |
| **SETD2** | 29072 | 3p21.31 | 0 | -1 | -1 | -1 | -1 |
| **STK11** | 6794 | 19p13.3 | 0 | -1 | -1 | -1 | -1 |
| **TSC2** | 7249 | 16p13.3 | 0 | -1 | -1 | -1 | -1 |
| **BRCA2** | 675 | 13q13.1 | 0 | -1 | -1 | -1 | -1 |
| **FAT4** | 79633 | 4q28.1 | 0 | -2 | -1 | -1 | -1 |
| **PRDM2** | 7799 | 1p36.21 | 0 | -2 | -1 | -1 | -1 |
| **POLE** | 5426 | 12q24.33 | 1 | -1 | -1 | -1 | -1 |
